# Supplementary material for: Ex-situ avian sex skews: determinants and implications for conservation
Source: PeerJ. 2025 Apr 18;13:e19312. doi: 10.7717/peerj.19312 (PMC12011015; doi:10.7717/peerj.19312)
Supplement: Supplemental Information 1 [file peerj-13-19312-s001.docx]

**Supplementary Table 1.**

Descriptive statistics showing mean proportion of males amongst 30 orders in global bird holdings where pink shading indicates female skew (≤ .494), green is at parity (= .495 – .504) and blue is a skew to male (≥ .505).

| Order | Common nomenclature | *N* | Mean  prop. males | *SD* |
| --- | --- | --- | --- | --- |
| Struthioniformes | Ostrich, cassowary, emu, kiwi, rhea and tinamou | 1210 | .469 | 0.342 |
| Cariamiformes | Seriemas | 107 | .487 | 0.351 |
| Accipitriformes | Hawks, eagles and kites | 2337 | .488 | 0.379 |
| Cathartiformes | New world vultures | 418 | .488 | 0.359 |
| Strigiformes | Owls | 2181 | .502 | 0.367 |
| Charadriiformes | Gulls, auks, sandpipers and plovers | 921 | .504 | 0.308 |
| Sphenisciformes | Penguins | 509 | .512 | 0.180 |
| Gruiformes | Cranes, rails, limpkins and allies | 1354 | .513 | 0.315 |
| Anseriformes | Ducks, geese, swans, screamers and magpie geese | 5884 | .516 | 0.312 |
| Musophagiformes | Turacos and quetzals | 534 | .526 | 0.329 |
| Bucerotiformes | Hornbills and hoopoes, wood hoopoes | 1023 | .528 | 0.325 |
| Falconiformes | Falcons and caracaras | 553 | .531 | 0.396 |
| Galliformes | Chickens, new world quail, guinea fowl, chachalacas, brush-turkeys and allies | 3192 | .531 | 0.316 |
| Pelecaniformes | Pelicans, herons, ibises and allies | 2107 | .538 | 0.298 |
| Ciconiiformes | Storks | 2392 | .539 | 0.306 |
| Coraciiformes | Kingfishers, bee-eaters, rollers, motmots and todies | 639 | .540 | 0.331 |
| Piciformes | Woodpeckers, honeyguides, toucans and barbets | 460 | .545 | 0.351 |
| Phoenicopteriformes | Flamingos | 572 | .551 | 0.195 |
| Psittaciformes | Parrots and cockatoos | 6621 | .553 | 0.339 |
| Passeriformes | Perching birds | 4323 | .556 | 0.333 |
| Otidiformes | Bustards, floricans and korhaans | 65 | .560 | 0.356 |
| Columbiformes | Pigeons and doves | 2005 | .565 | 0.308 |
| Caprimulgiformes | Nightjars, frogmouths, hummingbirds, owlet-nightjars, and oilbirds | 148 | .596 | 0.319 |
| Suliformes | Gannets, boobies, cormorants, anhingas and frigatebirds | 61 | .596 | 0.352 |
| Coliiformes | Mousebirds | 56 | .607 | 0.323 |
| Eurypygiformes | Sunbitterns and kagu | 99 | .607 | 0.323 |
| Trogoniformes | Trogons and quetzals | 21 | .613 | 0.325 |
| Cuculiformes | Cuckoos | 139 | .620 | 0.358 |
| Pterocliformes | Sandgrouse | 19 | .626 | 0.254 |
| Podicipediformes | Grebes | 14 | .657 | 0.367 |
