# Supplementary material for: Ex-situ avian sex skews: determinants and implications for conservation
Source: PeerJ. 2025 Apr 18;13:e19312. doi: 10.7717/peerj.19312 (PMC12011015; doi:10.7717/peerj.19312)
Supplement: Supplemental Information 2 — Asterisk represents a significant result. [file peerj-13-19312-s002.docx]

**Supplementary Table 2.**

Post hoc analysis for ANOVA test of between group difference in proportion of males within IUCN threat categories. Asterisk represents a significant result.

|  | Comparison | | |  | | | |
| --- | --- | --- | --- | --- | --- | --- | --- |
| Order | **IUCN** |  | **IUCN** | **Mean Difference** | ***df*** | ***t*** | **p_tukey_** |
| Columbiformes | Least Concern | - | Near Threatened | 0.01057 | 1999 | 0.647 | .987 |
|  |  | - | Vulnerable | 0.045 | 1999 | 2.001 | .341 |
|  |  | - | Critically Endangered | 0.040 | 1999 | 0.185 | 1 |
|  |  | - | Endangered | 0.014 | 1999 | 0.205 | 1 |
|  |  | - | Extinct in the Wild | -0.128 | 1999 | -2.597 | .098 |
|  | Near Threatened | - | Vulnerable | 0.034 | 1999 | 1.388 | .734 |
|  |  | - | Critically Endangered | 0.029 | 1999 | 0.136 | 1 |
|  |  | - | Endangered | 0.003 | 1999 | 0.051 | 1 |
|  |  | - | Extinct in the Wild | -0.138 | 1999 | -2.748 | .066 |
|  | Vulnerable | - | Critically Endangered | -0.004 | 1999 | -0.022 | 1 |
|  |  | - | Endangered | -0.031 | 1999 | -0.432 | .998 |
|  |  | - | Extinct in the Wild | -0.173 | 1999 | -3.281 | *.013 |
|  | Critically Endangered | - | Endangered | -0.026 | 1999 | -0.114 | 1 |
|  |  | - | Extinct in the Wild | -0.168 | 1999 | -0.756 | .975 |
|  | Endangered | - | Extinct in the Wild | -0.142 | 1999 | -1.692 | .537 |
|  | *Note.* Comparisons are based on estimated marginal means | | | | | | |
